# A Comparative Study of Physically Accurate Synthetic Shadow Datasets in Agricultural Settings with Human Activity

**DOI:** 10.3390/s24092737

**Published:** 2024-04-25

**Authors:** Mengchen Huang, Ruben Fernandez-Beltran, Ginés García-Mateos

**Affiliations:** Department of Computer Science and Systems, University of Murcia, 30100 Murcia, Spain; mengchen.huang@um.es (M.H.); rufernan@um.es (R.F.-B.)

**Keywords:** shadow segmentation, synthetic dataset, transfer learning, out of domain, computer vision

## Abstract

Shadow, a natural phenomenon resulting from the absence of light, plays a pivotal role in agriculture, particularly in processes such as photosynthesis in plants. Despite the availability of generic shadow datasets, many suffer from annotation errors and lack detailed representations of agricultural shadows with possible human activity inside, excluding those derived from satellite or drone views. In this paper, we present an evaluation of a synthetically generated top-down shadow segmentation dataset characterized by photorealistic rendering and accurate shadow masks. We aim to determine its efficacy compared to real-world datasets and assess how factors such as annotation quality and image domain influence neural network model training. To establish a baseline, we trained numerous baseline architectures and subsequently explored transfer learning using various freely available shadow datasets. We further evaluated the out-of-domain performance compared to the training set of other shadow datasets. Our findings suggest that AgroSegNet demonstrates competitive performance and is effective for transfer learning, particularly in domains similar to agriculture.

## 1. Introduction

In the realm of computer vision, extensive research has been devoted to the challenge of shadow detection and segmentation, which holds significant utility for various tasks. One such task is shadow removal, which streamlines numerous other computer vision problems. Equally compelling is the detection of scenarios where the absence of shadows is of interest, particularly in determining whether a specific area in an image is directly illuminated, often by the sun in outdoor settings. This capability proves valuable in applications such as the positioning of photovoltaic panels, where shadows can diminish the efficiency of solar modules, potentially leading to temperature-induced damage known as hot spots [1]. Additionally, it finds relevance in monitoring solar radiation for crop health, ensuring that plants receive adequate sunlight for photosynthesis, a crucial aspect of plant well-being. Thus, the broader implications of shadow detection extend beyond mere image processing, offering solutions to real-world challenges in diverse domains.

Shadow datasets serve various purposes. For general applications, they are utilized in tasks such as shadow segmentation, which aids in identifying shadows and image manipulation. Shadow removal is another common use, particularly valuable for image processing tasks. For example, removing shadows can enhance the performance of object detection models and augmented reality applications, allowing for the realism of virtual objects by overlaying and compositing shadows in software, thereby improving immersion.

Aerial satellite imagery shadow datasets have specific applications in urban planning, such as roof solar panel planning [2], analyzing the impact of buildings and structures on the surrounding environment and sunlight exposure. Remote sensing is another application, useful for terrain mapping by estimating the height of terrain through shadow analysis. Vegetation analysis is also facilitated by aerial shadow datasets, providing information about the density of vegetation cover by analyzing the uniformity of shadows.

Lastly, agricultural shadow datasets are valuable for crop monitoring through remote sensing to determine whether plants receive sufficient sunlight, which can affect parameters such as the Normalized Difference Vegetation Index (NDVI) [3], widely used for plant health monitoring.

In the context of our intended use case, also known as agrophotovoltaics [4,5], a field is equipped with motorized photovoltaic panels and cameras mounted on a support structure at approximately 3 m in height. Our primary objective is to balance the utilization of both solar radiation and crop growth simultaneously. This involves ensuring that plants receive sufficient sunlight for photosynthesis while harnessing excess sunlight for photovoltaic energy production. In some cases, introducing shade when plants are saturated with sunlight can improve crop yield, as demonstrated in studies [6]. We aim to achieve this balance through the detection of shadows using cameras, enabling us to adjust the position of motorized photovoltaic panels in future iterations to optimize solar radiation distribution.

To initiate data collection without the need to physically construct the system in an actual field, saving both time and costs, we suggest creating a virtual representation. This involves modeling a simulated field in 3D modeling software, providing greater flexibility compared to the real world. This virtual approach enables us to easily adjust lighting parameters and simulate various seasons and times, facilitating a more efficient analysis and more accurate ground truth data.

The challenges of shadow detection and removal have been extensively studied, with notable standard datasets contributing to the research. For shadow detection, the SBU dataset [7] is widely recognized, comprising approximately 5000 images featuring shadows across diverse scenes and photo types. Despite its significance, the dataset suffers from noisy annotations. Another prominent dataset is ISTD [8], addressing both shadow detection and removal, consisting of around 2000 images with cleaner annotations. However, the limitation of ISTD lies in its use of hand-taken photos and simpler environmental settings. More recently, the CUHK-Shadow dataset [9] has emerged, aiming to capture the complexity of shadows in the real world with more challenging environments, though it has copyright issues and gated access. Moreover, there is the Aerial Imagery Dataset for Shadow Detection (AISD) [10], which provides a unique perspective, particularly relevant for aerial imagery applications such as remote sensing. However, compared to other generic shadow datasets, the macroscale results in a different level of detail, which might not be desired for the intended application. AISD consists of around 500 pairs of top-down aerial images with manually labeled shadow masks. These masks are created by first selecting and filtering regions with clearly defined shadows from the source dataset, such as buildings and trees. The AISD is based on the Inria Aerial Image Labeling Dataset.

In the realm of synthetic datasets, the GTAV Shadow Removal Dataset [11] stands out as particularly notable. It leverages existing scenes from a video game, thereby saving time on modeling the environment. Another similarly innovative dataset is the Rendered Shadow Generation Dataset (RdSOBA) [12], which comprises a vast collection of shadow-object pairs constructed directly from a game engine, providing greater control over scenes. Both datasets employ a rendering technique based on rasterization, commonly used in video games due to its superior performance compared to ray tracing, which aims to simulate the realistic behavior of light rays. This approach sacrifices rendering speed for accuracy and realism in lighting. To obtain the ground truth data for shadow masks, the rendering pipeline is adjusted to disable shadows.

Moreover, works such as SynShadow [13] take a different approach by utilizing composite shadows. This method enables the generation of datasets on the fly by overlaying predefined shadow masks with shadow-free images from another dataset, such as USR [14].

Motivated by the observations above, we propose to advance shadow detection within specific domains, such as agricultural images, in contrast to shadow detection in images from different domains. This will be achieved by comparing the performance of baseline models and utilizing transfer learning across different datasets. Our work contributes to the field in two primary aspects.

First, we introduce AgroSegNet [15], an agricultural shadow detection dataset generated from a virtual scene, aimed at rectifying several deficiencies inherent in existing shadow datasets within the agricultural domain. These include the absence of datasets captured from a top-down camera perspective distinct from UAV or satellite views, scarcity of scenes featuring a camera positioned between crops and shadow-casting obstacles to simulate scenarios like PhotoVoltaic (PV) panels, inclusion of both self-shadows from plants and externally cast shadows, and the lack of high-accuracy shadow masks attributed to the challenge of annotating transparency in plant structures. This dataset comprises 50,000 top-down images along with corresponding masks generated by a ray-tracing renderer, ensuring the inclusion of physically accurate shadow masks. The scene includes the possible appearance of people, simulating workers doing their activity in the field. This allows its use in human activity detection and recognition systems.

Second, we establish a baseline evaluation model for AgroSegNet and provide a benchmark for assessing performance. Additionally, we explore the efficacy of simple transfer learning techniques between models trained on AgroSegNet and those generated from other datasets.

## 2. Materials and Methods

A large-scale dataset plays a crucial role in training a high-performance deep learning model. However, in our specific domain of shadow detection in agricultural settings with a top-down facing camera, there is a notable absence of a domain-specific shadow dataset. Therefore, we have chosen to investigate the utilization of a syntactical dataset. This approach aims to both save time and enhance annotation quality compared to using a traditional dataset.

### 2.1. Synthetic vs. Traditional

In contrast to the traditional method of manually capturing photos on-site, which poses challenges in terms of planning the location, timing, and obtaining permissions, a virtual approach streamlines the process. It involves modeling the scene using 3D computer graphics software, eliminating the need for physical setups and overcoming limitations associated with weather conditions and location restrictions. This includes factors like preparing equipment (e.g., tripod and camera) for stable angles. Moreover, considerations for weather, season, and time of day are crucial for real-life photography as they significantly impact lighting, shadows, and overall composition. The duration of the virtual approach varies based on scene complexity and the modeling software, ranging from a few days to several months, depending on whether an existing model is reused or created from scratch.

### 2.2. Preparing the Virtual Scene

For our virtual scene created for AgroSegNet, we utilized the powerful 3D modeling and rendering software Blender 3.6.4 (Blender Foundation, Amsterdam, Netherlands) [16]. The scene was meticulously crafted from scratch, integrating textures and models sourced from various online repositories. We carefully curated a selection of 7 plant models and 21 distractor models, including rocks, logs, shoes, bottles, and more. These elements were procedurally instanced and distributed in a grid pattern across a terrain sculpted to resemble a groove-like form. This deliberate arrangement aimed to infuse the scene with diversity and vibrant colors.

Furthermore, to imbue the environment with a sense of vitality, we incorporated 5 human models, each adopting randomized poses drawn from a pool of 14 predefined stances.

**Figure 1:**
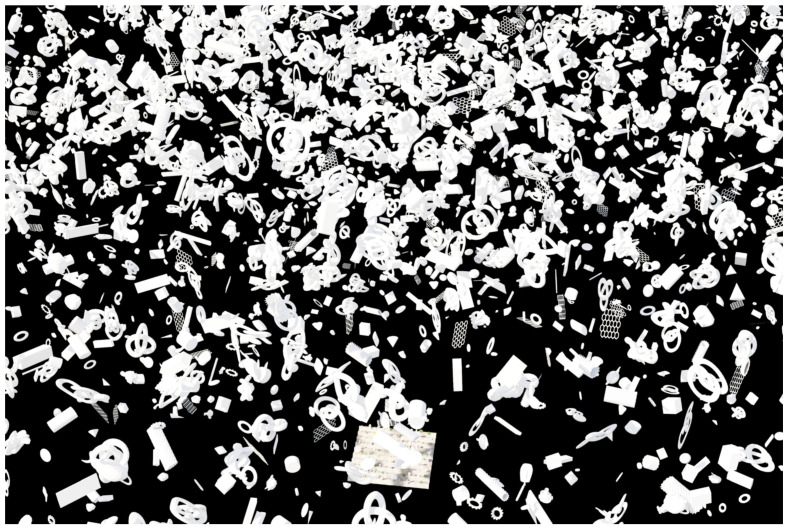
Shadow-caster obstacles positioned directly above the scene and camera.

**Figure 2:**
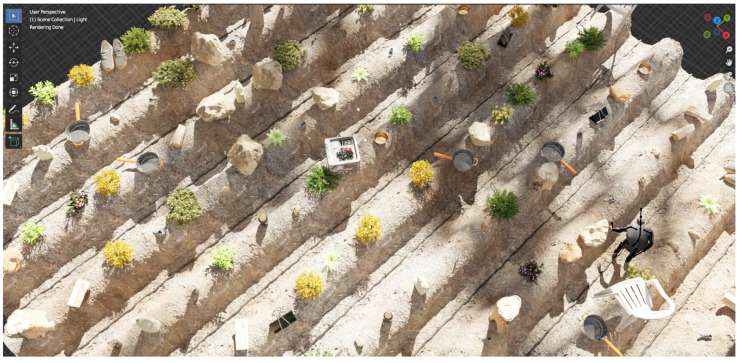
Preview render of the virtual scene in Blender. The dark spots are shadows cast by external shadow casters.

To enhance realism and simulate the interplay of light and shadow, we introduced a collection of obstacles representing external shadow casters. These obstacles, constructed from basic primitive shapes such as cubes, cones, honeycombs, and tori, were procedurally instanced and scattered beyond the camera’s view, positioned above the terrain shown in Figure 1. This technique simulated the presence of objects like clouds, poles, structures, and solar panels, enriching the visual complexity of the scene, as illustrated in Figure 2.

### 2.3. Lighting Setup

**Figure 3:**
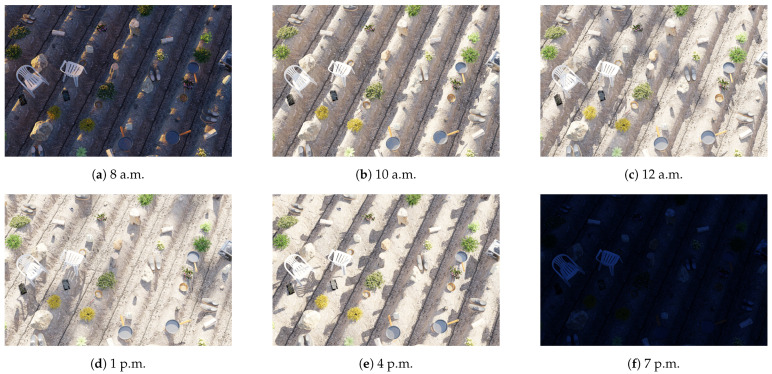
Example render of the same scene and camera position, but with varying times of day from 8 a.m. to 7 p.m., showcasing the differences in lighting conditions throughout the day.

For realistic lighting with accurate shadow and bounces, we opted for Cycle, a ray-tracing renderer, instead of Eevee, a rasterization renderer in Blender. This choice enabled better environment lighting. We utilized a sky texture generated with the Nishita [17,18,19] algorithm to simulate the colors of the sky, adjusting the color based on atmospheric parameters such as density of air molecules (Air), density of dust molecules and water droplets (Dust), and density of the ozone layer (Ozone) to simulate different atmospheric conditions. Additionally, we employed a plugin in Blender called *“Sun Position"* to simulate the rotation of the sun based on the timestamp and geolocation on Earth, using the Earth System Research Laboratory’s solar calculator [20]. This allowed us to control the sun disc on the Nishita sky texture, resulting in a procedural scene with realistic lighting, as illustrated in Figure 3.

### 2.4. Render Optimization

When capturing images in the physical world, the primary cost per image, excluding initial setup expenses, is attributed to human time. On the contrary, synthetic images generated in a 3D environment incur hardware-related costs, which translate to electricity expenses and equipment costs if computing hardware is rented. However, due to recent advances in denoising models in ray tracing, the time to render each image can be significantly reduced by decreasing the number of samples per render and utilizing a denoiser algorithm such as OpenImageDenoise [21]. This approach turns out to be more cost-efficient than relying solely on human labor, thereby enabling the collection of significantly larger datasets by another order of magnitude.

### 2.5. Calculating the Shadow Mask

**Figure 4:**
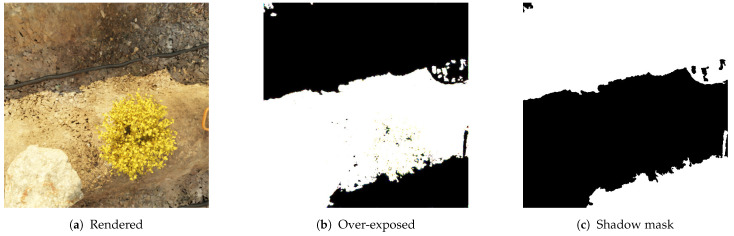
(**a**) Standard rendered image with realistic lighting. (**b**) Over-exposed version of (**a**), achieved by increasing the brightness of the sun and removing indirect lighting. (**c**) Shadow mask generated by post-processing (**b**), involving filtering to remove small contours and applying a threshold to convert the image into a binary mask.

The next step after acquiring the source image is annotating the shadow mask. This process replaces manual labeling of the dataset by hand or using tool-assisted methods, or applying heuristics to post-process the shadow mask [7]. In synthetic datasets, shadow masks are generated by adjusting lighting parameters, such as increasing the strength of the primary direct light source, typically representing the sun, while disabling environmental and indirect lighting (shown in Figure 4b). To convert these masks into binary form, further post-processing is conducted. This includes filtering contours with small areas to eliminate rendering artifacts caused by extreme lighting conditions, followed by applying a threshold to convert overexposed renders into shadow masks (illustrated in Figure 4c).

### 2.6. Procedural Generation

Moreover, a script has been developed utilizing Blender’s API to efficiently generate batches of datasets. To enhance diversity, the script dynamically adjusts the seed used for random procedural placement of plants, distractors, and obstacles. Furthermore, it modifies atmospheric settings such as air, dust, and ozone used by the Nishita algorithm. The script also randomizes location and temporal data for calculating the sun’s position, camera’s position, focal length (between 47 and 53 mm), and rotation. Additionally, it dynamically generates terrain textures by compositing multiple textures with various Perlin noises.

### 2.7. Evaluation Methods

To evaluate our dataset, we employed multiple baseline models using commonly utilized encoders such as *ResNet50* [22] and *EfficientNet_B5* [23], along with segmentation decoders like *U-Net* [24], *U-Net++* [25], and *PSPNet* [26]. These models were trained to establish a baseline performance.

Following the establishment of the baseline, we conducted a benchmarking exercise to measure the dissimilarity between our dataset and others. This involved initially training the models using the training set of our source dataset, and subsequently testing them against the test sets of other datasets.

To assess the performance of our models, we chose to employ widely recognized metrics commonly used in machine learning. These include Dice Loss, measuring the similarity and F-score ($F_{1}$), which represents the harmonic mean of precision and recall, ranging from 0 to 1 where 1.0 signifies perfect precision and recall. Additionally, we utilized metrics commonly employed in segmentation tasks, such as Intersection over Union (IoU, also known as Jaccard’s Index). Lastly, we evaluated using Balanced Error Rate (BER), a widely used metric for shadow detection [7,8,9], where lower values denote superior performance.

(1) $$\mathrm{Dice}\;\mathrm{Loss}\left(A,B\right)=1-2\cdot \frac{\left|A\cap B\right|}{\left|A|+|B\right|}$$

(2) $$F_{1}=2\cdot \frac{\mathrm{precision}\cdot \mathrm{recall}}{\mathrm{precision}+\mathrm{recall}}=\frac{2\cdot TP}{2\cdot TP+FP+FN}$$

(3) $$\mathrm{IoU}\left(A,B\right)=\frac{\left|A\cap B\right|}{\left|A\cup B\right|}$$

For the specific scenario of binary classification, the Intersection over Union (IoU) metric can be defined as

(4) $$\mathrm{IoU}=\frac{TP}{TP+FN+FP}$$

(5) $$BER=\left(1-\frac{1}{2}\left(\frac{TP}{TP+FN}+\frac{TN}{TN+FP}\right)\right)\cdot 100$$

Finally, to assess the potential for utilizing our dataset as a base weight for transfer learning to expedite learning in the early epochs, we conducted a simple transfer learning experiment, without freezing any layers, with the different datasets mentioned before.

**Figure 5:**
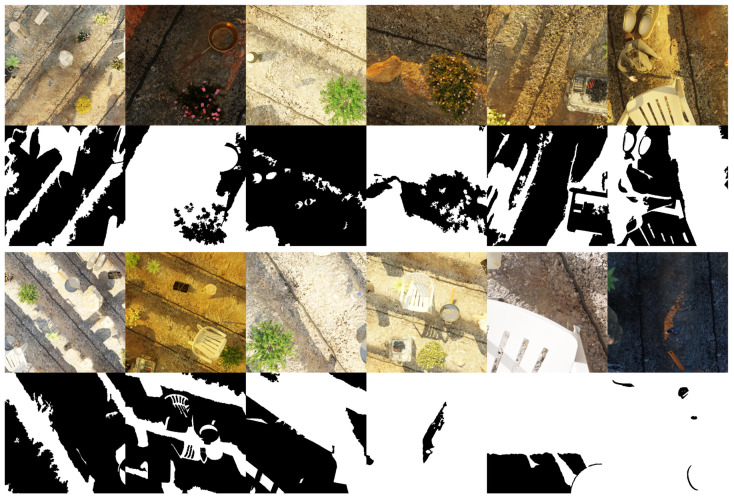
Preview of AgroSegNet shadow dataset.

The final dataset generated, AgroSegNet (previewed in Figure 5), consists of 50,000 pairs of images, comprising rendered images and shadow masks. These were divided into 40,000 pairs for the training set and the remaining 10,000 pairs for the test set. Due to the large volume of data (>25 GiB), Hugging Face was selected as the data repository, due to its unlimited storage, fast upload/download speeds, streamlined Python data loader, and built-in data viewer, which allows for previewing the dataset directly on the website without the need to download the entire dataset. Furthermore, a smaller version containing 125,000 image pairs is also available for experimentation.

The dataset is available at: https://www.doi.org/10.57967/hf/1652 (accessed on 4 February) [15].

## 3. Results

In this section, first, we present baseline results for AgroSegNet by training with different architecture by combining different backbones and segmentation heads, continued with a cross-dataset evaluation by testing models trained by each dataset with other datasets. Finally, we discuss the potential impact of transfer learning.

### 3.1. Baseline Model for AgroSegNet

**Figure 6:**
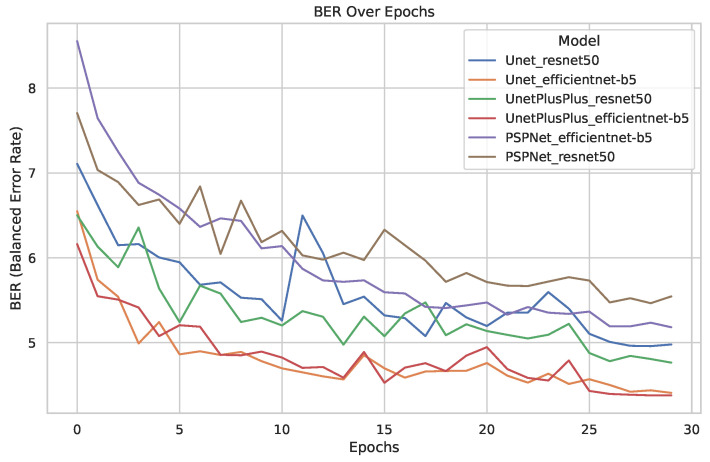
Plot illustrating test error (measured in Balanced Error Rate, BER) across 30 epochs for different models on AgroSegNet.

The training of various backbone architectures and segmentation heads to establish a baseline was conducted over 30 epochs. We used a batch size of eight, an initial learning rate of 0.0001, which was decreased to 0.00001 after 25 epochs, the Adam optimizer, and sigmoid activation. The evaluation of the models from the last epoch is also presented in Table 1, which contains additional metrics at epoch 30, such as Dice Loss, which is mainly used as a loss function in training that focuses on the similarity between the two masks, penalizing dissimilarities, while IoU measures the ratio of intersection to union, giving a sense of how much the predicted mask covers the ground truth mask. In contrast to Dice Loss and IoU, which are unbalanced metrics, both F-score and Balanced Error Rate (BER) are balanced, where F-score focuses on recall and precision and BER instead focuses on false positives and false negatives. Figure 6 illustrates the training process.

### 3.2. Cross-Dataset Evaluation

**Figure 7:**
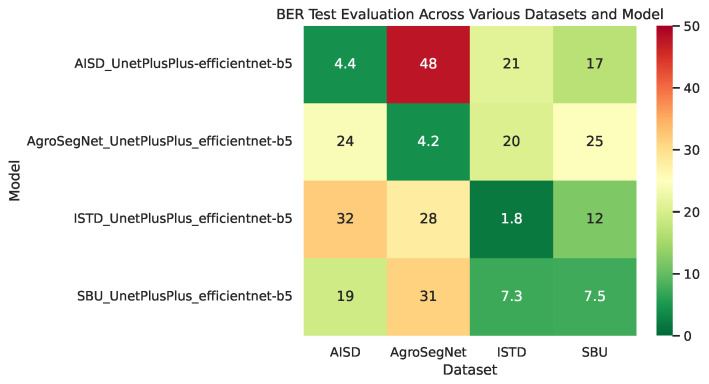
Balanced Error Rates (BER) across various datasets and models.

**Figure 8:**
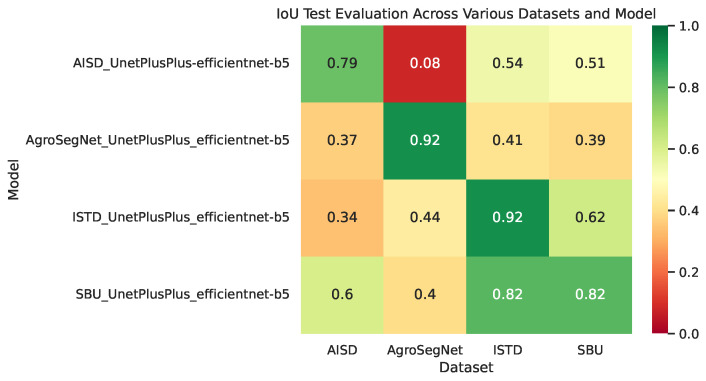
IoU across various datasets and models.

In order to evaluate the similarity between the datasets, multiple models were trained from scratch with different collections. For each model, Unet++ and EfficientNet-b5 architectures were utilized, together with a batch size of eight with a 0.0001 learning rate, and the Adam optimizer with a sigmoid activation for 20 epochs. Then, cross-evaluations between different datasets and models trained over each collection were zero-shot tested with several metrics, shown in Figure 7 for BER and Figure 8 for IoU, which can be interpreted as the ratio of overlap between prediction mask and ground truth. For datasets with irregular image dimensions, a preprocessing of cropping and padding to a size of 512 × 512 facilitated batch training. To measure the dataset and model’s overall performance, Table 2 was created, where the mean metrics for each row or column of Figure 7 and Figure 8 are displayed.

**Figure 9:**
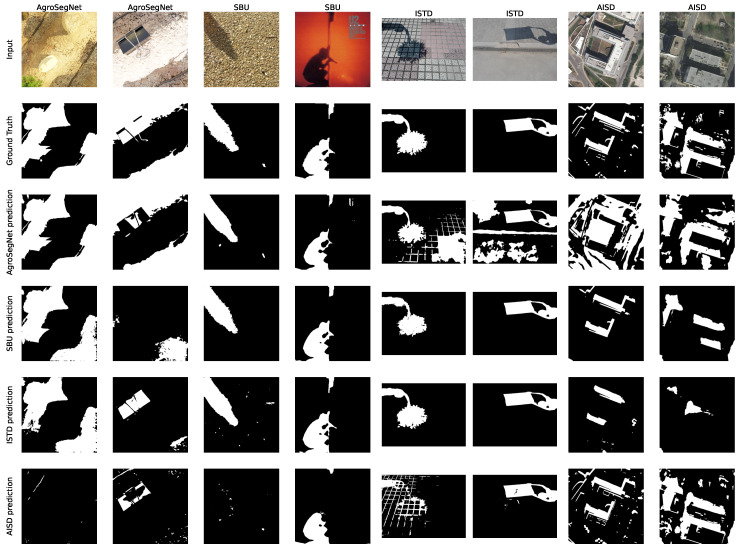
Comparison of predictions by different models on various datasets.

To further explore the results visually, we generated Figure 9 by randomly selecting two images from each dataset. Each row in the figure depicts the predictions generated by the respective models.

### 3.3. Transfer Learning

**Figure 10:**
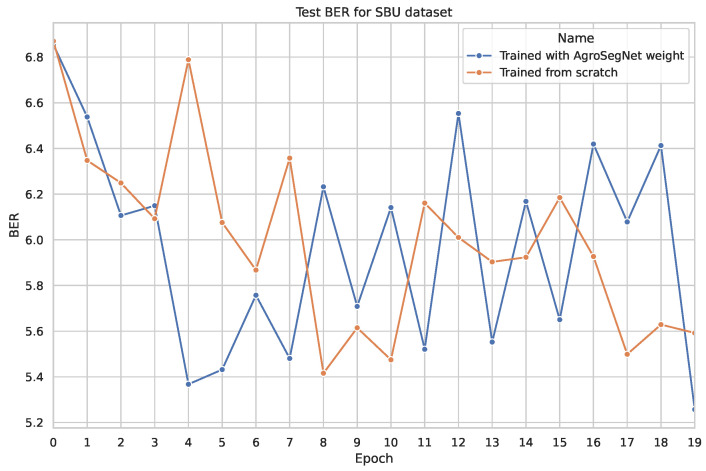
Comparing test evaluation across epochs: transfer learning vs. standard training on SBU dataset.

**Figure 11:**
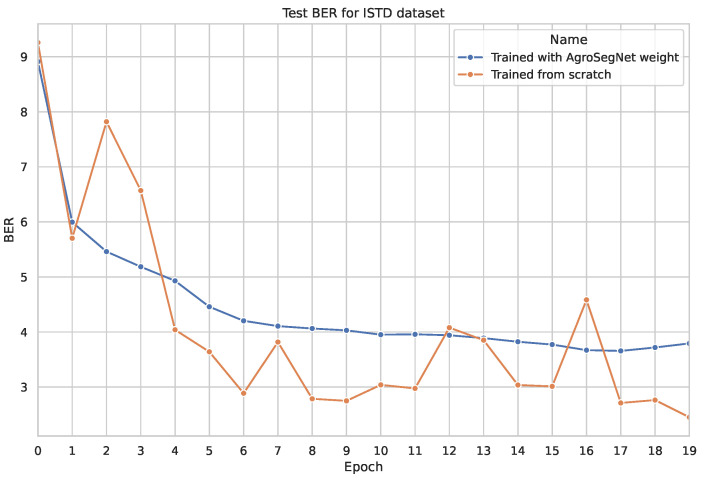
Comparing test evaluation across epochs: transfer learning vs. standard training on ISTD dataset.

**Figure 12:**
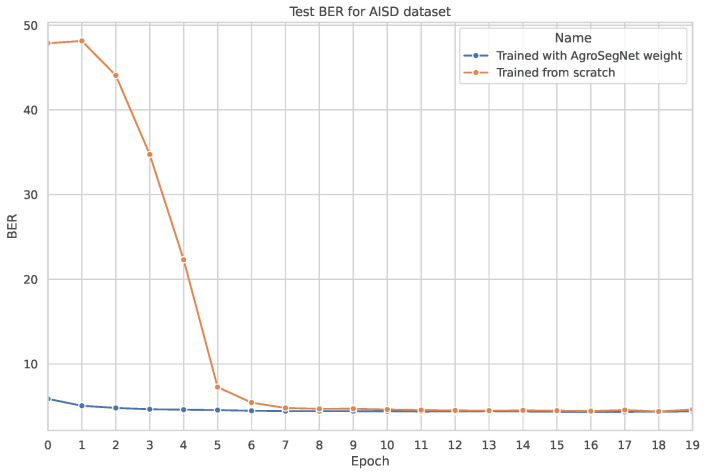
Comparing test evaluation across epochs: transfer learning vs. standard training on AISD dataset.

**Figure 13:**
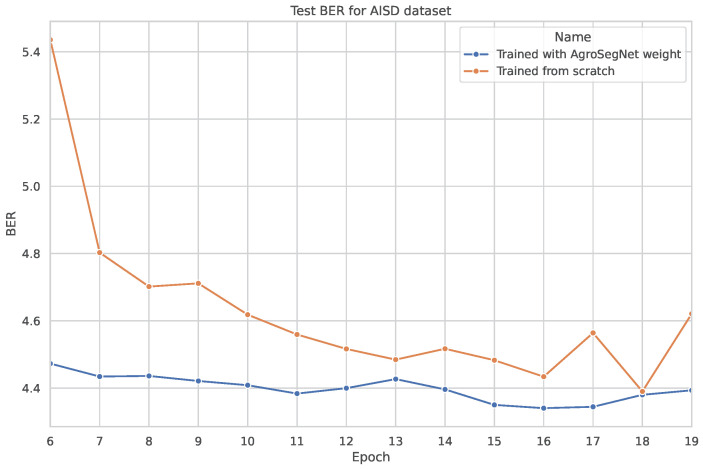
Same plot as Figure 12, but zoomed in starting from epoch 6.

A simple transfer learning experiment was conducted to examine whether a synthetic dataset could be utilized as a base for other shadow datasets. The base model was trained for 20 epochs, with both the settings for the base model and the dataset preprocessing identical to those described in Section 3.2. For the transfer learning phase, the base model was further trained using novel datasets without freezing any layers, employing a learning rate of 0.0002 for an additional 20 epochs. Subsequently, it was tested against the same novel dataset. The results are depicted in Figure 10 for SBU, Figure 11 for ISTD, and Figure 12 and Figure 13 for AISD. Figure 13 starts at epoch 6 with a narrower y-axis range for better visualization. A table with the numerical results at epochs 5 and 20 is shown at Table 3.

## 4. Discussion

### 4.1. Baseline Model for AgroSegNet

Based on the results depicted in Figure 6 to establish a baseline, several conclusions can be drawn. Firstly, there was a significant variance between backbones, with EfficientNet-b5 demonstrating notably superior performance compared to resnet50. Moreover, the disparity between Unet and Unet++ was marginal. Surprisingly, the performance of PSPNet, even with a superior backbone, was inferior to that of Unet with a less advanced backbone. Viewing Table 1, which contains an additional metric at epoch 30, such Dice Loss was mainly used as a loss function for training that focused on the similarity between the two masks, penalizing dissimilarities, while IoU measured the ratio of intersection to union, giving a sense of how much the predicted mask covered the ground truth mask. Additionally, even without fine-tuning, the models exhibited a reasonable performance of approximately 4.37 BER.

### 4.2. Cross-Dataset Evaluation

Followed the analysis of Figure 7 and Figure 8 to examine the similarity and difference between datasets, it is evident that ISTD and SBU share some similarities, showing less evaluation error between them compared to other datasets. However, SBU poses greater challenges, indicated by a significantly higher evaluation error of 7.5 BER along the diagonal (reflecting evaluation using the same dataset used in training), in contrast to the lower error of 1.8 BER observed in ISTD. This variance could potentially be attributed to SBU’s noisier and more diverse shadow masks compared to those of ISTD. Similarly, AgroSegNet and AISD display comparable behaviors, albeit with higher error rates. Intriguingly, when trained with ISTD, AgroSegNet performed more poorly than AISD, whereas with SBU, the opposite was observed, indicating better performance with AISD than AgroSegNet.

In Figure 7, which displays the Balanced Error Rate (BER) across different datasets and models, a BER below 25 suggests some correlation, while a BER below 12.5 indicates good performance. A BER below 5 indicates a very strong correlation. Notably, state-of-the-art models tailored for shadow detection typically achieve a BER around 3 for SBU [27,28,29] and between 1 and 2 for ISTD [27,28,29]. Despite not being specifically designed for shadow detection, our choice of architecture, UnetPlusPlus and EfficientNet-b5, performed reasonably well, particularly achieving a BER of 1.8 for ISTD.

Moving on to Figure 8, which presents Intersection over Union (IoU) across various datasets and models, IoU greater than 0.5 suggests some correlation, while IoU greater than 0.75 indicates strong correlation. Very strong correlation is inferred when IoU exceeds 0.9. It is worth noting that IoU is not a balanced metric; it heavily depends on the shadow ratio of the dataset. This dependency makes it less comparable between different datasets. However, IoU’s advantage lies in its straightforward interpretation: it measures the overlap ratio between prediction masks and ground truth.

In retrospect, models trained with AgroSegNet may seem inferior to those trained with ISTD or SBU, due to higher error. However, this is attributed to the close domain alignment between ISTD and SBU, both serving as general-purpose shadow datasets, while AISD and AgroSegNet are more domain-specific, tailored for aerial satellite imagery and top-down agriculture settings, respectively. Referring to Table 2, it is evident that for model evaluation, the AISD-trained model exhibits the poorest performance, possibly due to AISD’s distinct bias as the most domain-specific dataset, while SBU demonstrates the highest adaptivity among the four models, with AgroSegNet and ISTD falling in between.

Regarding the mean metrics shown in Table 2, the results indicate varying difficulty levels, with ISTD being the easiest and AgroSegNet the most challenging. This discrepancy can be attributed to factors such as differences in shadow detail levels and the quantity of shadow present. Notably, ISTD mainly comprises single or two large shadow patches, whereas AgroSegNet features much more complex shadows. Additionally, variations in shadow mask criteria contribute to the difficulty, as AgroSegNet considers soft shadows as valid, adding complexity.

When examining Figure 9, we can discern the behavior of various datasets supporting our findings. For instance, we note that the masks predicted by SBU and ISTD exhibit remarkable similarity. However, they falter when applied to more intricate datasets like AgroSegNet and AISD, as they tend to overlook finer details, as evidenced by the second examples in both the AgroSegNet and AISD datasets. In contrast, models trained with AgroSegNet and AISD data demonstrate a propensity for capturing these finer nuances. This is evident in the first example of the ISTD dataset, where both the AgroSegNet and the AISD models erroneously label the dark tile spacing as shadow. Notably, models trained with datasets other than AgroSegNet struggled to approximate the intricacies of the second example in the AgroSegNet dataset, with AISD-trained models performing particularly poorly, failing even with the first example.

Despite being a fully synthetic dataset, AgroSegNet performs comparably to other real-world datasets, with trained models exhibiting similar performance, on average. However, it presents a greater challenge for models trained on other datasets, highlighting its adaptability to unknown domains while posing increased complexity. This underscores the significance of domain-specific datasets.

### 4.3. Transfer Learning

For the transfer learning experiment, Figure 10, depicts the testing error for SBU with and without utilizing models trained with AgroSegNet as a base for transfer learning. It is observed that utilizing AgroSegNet weights yielded significantly lower test errors before epoch 5 compared to training from scratch. However, after epoch 5, the results for both models become very similar and noisy, likely due to annotation errors in SBU’s masks, leading to reduced confidence and sensitivity to minimal weight changes during training.

Similarly, in the experiment with ISTD shown in Figure 11, transfer learning resulted in more stable and less noisy testing errors compared to training from scratch, although it performed poorly compared to models without transfer learning. This is attributed to the simplicity of shadows with straight edges present in ISTD, contrasting with the soft and more complex shadow shapes of AgroSegNet.

Analyzing the results presented in Table 3 confirms that for the SBU and AISD datasets, early epochs, such as epoch 5, demonstrate improved performance with transfer learning. However, there is a minimal difference observed at later epochs, such as epoch 20.

Finally, concerning AISD, as depicted in Figure 12, a significant difference is observed between training from scratch and utilizing transfer learning, particularly in the early epochs. However, starting from epoch 6, both models begin to converge around 4.5 BER. For a more detailed comparison, Figure 13 illustrates that the model with transfer learning consistently outperforms the model without transfer learning in all epochs. This is likely due to the inherent similarities between AgroSegNet and AISD, both being top-down view shadow datasets, sharing some inherent biases.

## 5. Conclusions

In summary, AgroSegNet is a large-scale, fully synthetic shadow segmentation dataset designed specifically for agricultural settings with human activity. It features physically accurate shadow masks generated through virtual scenes and 3D rendering, giving it a competitive edge compared to other real-world shadow datasets. Taking into account the differences in target domains, AgroSegNet is more similar to top-down shadow datasets such as AISD than to general-purpose shadow datasets such as ISTD or SBU. This characteristic makes it particularly useful for transfer learning, especially for applications that involve analogous data. We anticipate that this adaptability will enhance shadow segmentation models for agricultural applications, particularly through the incorporation of real-field images and fine-tuning via transfer learning in future research endeavors.

Another avenue of exploration involves expanding our virtual scenes by incorporating additional plant types and ground layouts. This expansion aims to enhance dataset diversity and generate masks with varying attributes such as depth, class, and instance segmentation. Leveraging our virtual scene approach, incorporating these elements is relatively straightforward. We believe that this extension will further enrich the dataset’s utility and broaden its applicability in agricultural shadow segmentation tasks.

## Figures and Tables

**Table 1:** Performance comparison of different architectures after 30 training epochs using AgroSegNet dataset.

Encoder	Models	Dice Loss@30	IoU@30	*^F^1@30*	BER@30
ResNet50	Unet	0.051757	0.907921	0.948176	4.979060
EfficientNet-b5		0.045990	0.917021	0.953940	4.408404
ResNet50	**Unet++**	0.049745	0.911195	0.950185	4.764656
**EfficientNet-b5**		**0.045523**	**0.917817**	**0.954407**	**4.379995**
ResNet50	PSPNet	0.460912	0.887085	0.935562	5.545524
EfficientNet-b5		0.460150	0.893096	0.940081	5.181897

Best performers are highlighted in bold.

**Table 2:** Mean BER and IoU for each dataset and model. The best-performing model is highlighted in bold, while the most challenging dataset is denoted in bold.

Type	Dataset	Mean BER	Mean IoU
Model	AISD 22.565771	0.480851	
	AgroSegNet	18.380684	0.521470
	ISTD	18.408633	0.579932
	SBU	**16.299331 **	**0.659962**
Dataset	AISD	19.929658	0.524968
	AgroSegNet	**22.304499**	**0.459299**
	ISTD	12.655474	0.672963
	SBU	15.314512	0.584985

**Table 3:** Comparison of test evaluation metrics at two epochs (5 and 20) for various datasets using transfer learning and training from scratch.

Dataset	Method	BER@5	IoU@5	BER@20	IoU@20
SBU	with transfer learning	**5.367644**	**0.775531**	**5.257161**	0.791660
	from scratch	6.788574	0.781911	5.592205	**0.794267**
ISTD	with transfer learning	4.929123	0.764420	3.792633	0.811411
	from scratch	**4.039957**	**0.828133**	**2.452189**	**0.890311**
AISD	with transfer learning	**4.599337**	**0.779643**	**4.393424**	**0.804452**
	from scratch	22.304499	0.523356	4.620904	0.810667

Best performers are highlighted in bold.

## Data Availability

The original data presented in the study are openly available in HuggingFace at https://huggingface.co/datasets/Menchen/AgroSegNet (accessed on 4 February 2024).
